# DNA Helicase-SSB Interactions Critical to the Regression and Restart of Stalled DNA Replication Forks in *Escherichia coli*

**DOI:** 10.3390/genes11050471

**Published:** 2020-04-26

**Authors:** Piero R. Bianco

**Affiliations:** Center for Single Molecule Biophysics, University at Buffalo, SUNY, Buffalo, NY 14221, USA; pbianco@buffalo.edu; Tel.: +(716)-829-2599

**Keywords:** RecG, single-strand binding protein (SSB), Stalled DNA replication fork, DNA repair, DNA replication, helicase, atomic force microscopy, OB-fold, SH3 domain, PXXP motif

## Abstract

In *Escherichia coli,* DNA replication forks stall on average once per cell cycle. When this occurs, replisome components disengage from the DNA, exposing an intact, or nearly intact fork. Consequently, the fork structure must be regressed away from the initial impediment so that repair can occur. Regression is catalyzed by the powerful, monomeric DNA helicase, RecG. During this reaction, the enzyme couples unwinding of fork arms to rewinding of duplex DNA resulting in the formation of a Holliday junction. RecG works against large opposing forces enabling it to clear the fork of bound proteins. Following subsequent processing of the extruded junction, the PriA helicase mediates reloading of the replicative helicase DnaB leading to the resumption of DNA replication. The single-strand binding protein (SSB) plays a key role in mediating PriA and RecG functions at forks. It binds to each enzyme via linker/OB-fold interactions and controls helicase-fork loading sites in a substrate-dependent manner that involves helicase remodeling. Finally, it is displaced by RecG during fork regression. The intimate and dynamic SSB-helicase interactions play key roles in ensuring fork regression and DNA replication restart.

## 1. Introduction

The precise duplication of the genome relies on the DNA replication, DNA repair and genetic recombination machinery working closely together [1,2,3,4,5]. This complex interplay is required as the advancing replisomes frequently encounter roadblocks that have the potential to stall or collapse replication forks [6,7]. In *Escherichia coli*, forks stall on average at least once per cell cycle and require restart. The types of obstructions to fork progression include proteins bound to the parental duplex, non-coding lesions in the template DNA, unusual secondary structures that arise in the DNA and either single- or double-strand breaks [3,8,9,10,11,12]. Each of these roadblocks to DNA replication can use different subsets of repair enzymes and this is highlighted by the varied recombination and repair gene requirements for fork rescue [9,13,14,15,16,17]. For example, when the replisome encounters a nick in the leading strand, the structure of the fork will collapse and require the processing of the nascent double-strand break by RecBCD followed by strand invasion catalyzed by RecA. This results in the formation of a displacement loop that is then used to reload the replicative helicase DnaB leading to the restart of DNA replication [15].

**Figure 1:**
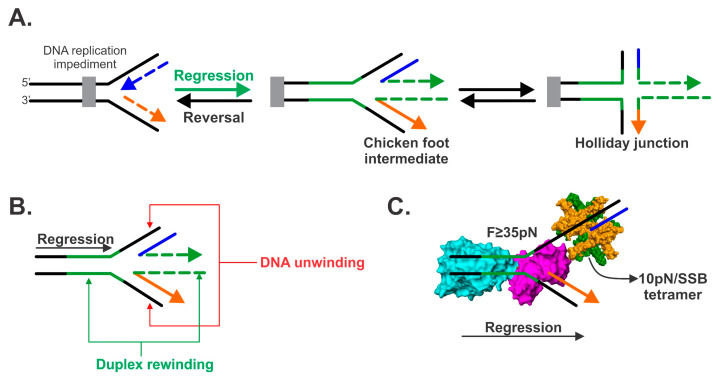
Fork regression. (**A**). Fork regression occurs in a direction opposite to that of DNA replication. An impeded fork is shown with the nascent leading and lagging strands colored blue and orange, respectively. Fork regression involves unwinding of these nascent duplex regions, concomitant with the extrusion of two duplex regions colored in green. The resulting DNA structure is known as the “chicken foot” and is structurally similar to a Holliday junction. Fork reversal (indicated by the balck arrow) results in leftward movement of the fork and an occur only once regression and subsequent impediment repair have taken palce. (**B**). Fork regression requires that DNA unwinding be coupled to duplex rewinding. (**C**). The RecG DNA helicase is shown in the process of fork regression where it couples DNA unwinding to duplex rewinding and displacement of the single-strand binding protein (SSB) protein. RecG and SSB are represented as Connolly surfaces. The wedge domain of RecG is colored purple and the helicase domains in cyan. SSB monomers are colored orange and green.

In contrast, when replisomes encounter an impediment such as a dsDNA crosslink or bound proteins such as RNA polymerase or mutant methyltransferases, a stalled DNA replication fork is formed [18]. A stalled fork can be directly restarted or reversed (regressed; Figure 1 and [7,9,13,19,20,21,22,23]). Consequently, regression occurs in a direction opposite to that of replication, with the fork actively moved away from the site of damage to a region where the nascent, replicated genome is unharmed. This enables the repair machinery to access the obstruction and facilitate repair. This process is analogous to the clearing of train tracks following a collision and possible derailment.

Which replisome proteins are disengaged from the DNA [24,25]? At a minimum, this must be DnaB because of the requirement for the restart DNA helicase, PriA [26,27,28]. However, due to the ability of polymerases to rapidly turn over, it is conceivable that these components, as well as others, might disengage as well [29,30]. However, protein disengagement may not always occur as recent work from the Marians group has shown [31]. In this study, they demonstrated that the replisome remains stably associated with the fork after a collision with a leading-strand template lesion. Leading-strand DNA synthesis was then reinitiated downstream of the damage in a reaction that is independent of any of the known replication-restart proteins.

If one or more components of the replisome disengage from the DNA, then replication cannot progress and the fork becomes stalled. Regression of the stalled DNA replication fork likely occurs (Figure 1A). This can be driven by accumulated positive superhelical tension as shown by the Cozzarelli group [32,33]. It could also be catalyzed by several proteins including the recombinase RecA, the DNA helicase RecG and the resolvase RuvAB [34,35,36,37,38,39]. However, the evidence overwhelmingly demonstrates that RecG catalyzes fork regression as explained below.

RecA is unlikely to be involved in fork regression where either the leading or lagging strands contain exposed ssDNA. This follows because RecA is inhibited by a single-strand binding protein (SSB) [38]. However, if DNA damage is present in the parental duplex DNA ahead of the fork, RecA could nucleate nucleoprotein filaments at the site of damage, to which it binds with enhanced affinity relative to undamaged DNA [40]. Filament extension from the site of damage towards the fork could in principle move the fork in the regression direction as the leading strand arm is encompassed by the growing filament. Alternatively, fork regression could progress via a multi-step, DNA strand exchange reaction [38].

For many years a conundrum existed in the fork regression field as it was thought that the branch-specific DNA helicases RecG or RuvAB could each catalyze this reaction. This conundrum was resolved using a combination of bulk-phase biochemical and single-molecule approaches. Studies show that RecG outcompetes an 80-fold excess of RuvAB at forks; that RecG is assisted in fork regression by the single-strand DNA binding protein (SSB) whereas RuvAB is inhibited; that SSB loads RecG onto forks and in the process remodels the helicase and, once bound to the fork, RecG catalyzes an efficient regression reaction [41,42,43,44]. Here, the unique attributes of the specialized DNA helicase RecG come into play: the enzyme couples DNA unwinding to rewinding to generate sufficient force so that obstacles bound to either the leading or lagging strand arms are readily displaced [24].

Once the fork has been regressed and repair of the impediment taken place, the PriA DNA helicase takes center stage [45]. Its primary role is to mediate the loading of the replicative helicase, DnaB onto the DNA resulting in the restart of replication. While PriA can bind to forks on its own, binding is mediated by SSB, with the helicase being remodeled in the process similar to what is observed for RecG [46,47,48].

## 2. Fork Regression Defined

The reaction known as fork reversal or fork regression is a unique reaction that requires a specialized DNA helicase (Figure 1A and see Section 3.1). In this reaction, the axis of the fork is moved away from the impediment in a direction opposite to that of replisome movement. Upon close examination of the movement of the DNA junction, it is immediately apparent that both DNA unwinding and duplex rewinding must occur concurrently (Figure 1B). That is to say, the nascent leading and lagging arms of the fork must be unwound, and the duplex parental DNA rewound. Additionally, and if the nascent arms contain sufficient regions of complementary dsDNA, an additional region of duplex rewinding will be observed, and this is extruded both in the wake of the enzyme and also ahead of the regressing fork. When viewed in this manner, the resulting structure resembles the foot of chicken, which has three toes. Not surprisingly, this intermediate is known as the “chicken foot intermediate” (Figure 1A and [34]). Simply by manipulating the arms of the regressed fork to the vertical position and by the inclusion of the parental duplex in the picture, it is plain to see that the resulting structure is also a Holliday junction, the primary substrate for the RuvAB resolvase [49]. Formation of the Holliday junction intermediate is central to many models proposed for fork rescue.

In addition to being able to both unwind and rewind DNA strands; the enzyme catalyzing fork regression must be able to clear the DNA near the fork of bound protein obstacles (Figure 1C). Included here are proteins bound to ssDNA gaps such as SSB and which requires 10 pN of force for displacement, and proteins that had bound to nascent duplex arms behind the advancing fork such as repressors or even nucleoid-associated proteins. As demonstrated below, a single, monomeric enzyme, the RecG DNA helicase, possesses all of the activities alluded to above and can work against forces >35 picoNewtons (pN) of force during regression.

## 3. The Protein Players

### 3.1. SSB—The Mediator of DNA Transactions at Forks

The single-stranded DNA binding protein (SSB) is essential to all aspects of DNA metabolism in *Escherichia coli* [50,51,52,53,54]. SSB has dual roles that are interconnected. First and as its name indicates, the protein binds to and stabilizes single-stranded DNA (ssDNA) intermediates generated during DNA processing. Second, it binds to as many as seventeen proteins temporally and spatially, to both store and target enzymes to the DNA when needed [46,55]. These roles are connected via the linker domain of the protein binding to OB-folds present in both SSB and its binding partners, as explained below.

**Figure 2:**
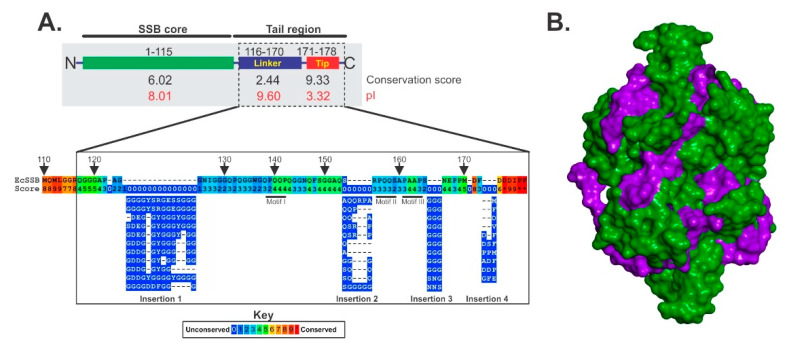
Organization of the SSB protein. (**A**), Schematic of SSB divided into the core and tail regions by proteolytic cleavage. The conservation scores for each region were calculated from alignments using Praline [61]. The pI of each region is shown in red and was calculated using the ProtParam tool of Expasy [62]. The pI of the intact protein is 5.44. (**B**), The SSB tetramer is intimately associated with ssDNA. The image was generated using PDB file 1EYG [58]. The DNA is colored purple and the tetramer, is colored green. Both protein and DNA are represented as Connolly surfaces.

SSB exists as a homo-tetramer with a monomer MW of 18,843 Da [56]. Each monomer is divided into two domains defined by proteolytic cleavage: an N-terminal domain comprising the first 115 residues and a C-terminal tail spanning residues 116–178, which includes the linker and acidic tip (Figure 2A and [57]). The N-terminal domains are responsible for tetramer formation and binding to ssDNA, which is mediated by the four OB-folds in the tetramer. Here, ssDNA binding by the OB-folds results in the wrapping of the polynucleotide around the SSB tetramer (Figure 2B and [58,59]). In addition, the OB-fold is also responsible for binding to the linker domain of nearby SSB tetramers as explained below [60]. Molecular modeling was used to show how the linker could bind to the OB-fold (Figure 4, right inset).

The disordered, C-terminal SSB tail can be further subdivided into two regions: a sequence of approximately 50 amino acids that has been called the intrinsically disordered linker (IDL) or linker, which has a pI of 9.6 (Figure 2A and [50,54,63,64]). The overall sequence conservation of the linker is poor and this is due to the presence of one to four insertions of different lengths affecting the overall alignment [64]) This is immediately followed by the acidic tip or tip, which is the last 8–10 residues of SSB. This region is very well conserved and is overall, acidic with a pI of 3.32 [50,63]. The C-terminal tail is essential for binding to partner proteins, which are collectively known as the “SSB-interactome” and include (not the complete list) SSB itself, Exonuclease I, Alkylation protein B, the χ-subunit of DNA polymerase, DnaG, RecO, uracil glycosylase, topoisomerase III and the PriA, RecG and RecQ DNA helicases [55,65,66].

Historically, the acidic tip was thought to be the primary and only, protein–protein interaction domain of SSB (for review see [50]). Surprisingly, studies using intact proteins show that the acidic tip is not required for binding at all and that binding is mediated by the linker [60,67,68,69]. Instead, the acidic tip functions to regulate the structure of the C-terminal domain using long-range electrostatic effects [70]. These effects ensure that the linker does not associate with the tetramer from which it emanates, making it available for partner binding. These interactions involve the linker of SSB binding to an OB-fold in the partner as explained in a subsequent paragraph.

As the tip is proposed to regulate the structure of the C-terminal tail of SSB, it was not surprising that when the acidic tip is mutated or deleted, the linker collapses back onto the SSB OB-fold, thereby inactivating the protein [63]. Mutant linker/OB-fold binding is rescued by high affinity, ssDNA-binding, which results in a conformational change in SSB that exposes the C-termini and makes the linkers available for partner binding [65,71]. Consistent, the affinity of SSB for the chi subunit of DNA polymerase and separately for PriA, increased when SSB was prebound to ssDNA [65]. Further support of the collapsing of mutant C-termini back onto the SSB tetramer, came from a recent atomic force microscopy study, which showed that the volume of wild type SSB is 3-fold higher than that of SSBΔC8, a mutant that lacks the acidic tip [48].

**Figure 3:**
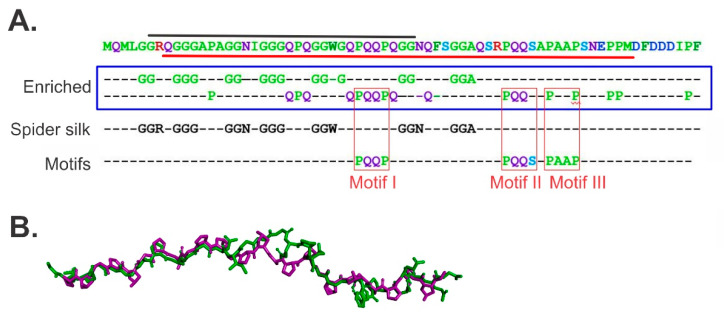
The linker domain of SSB contains sequence elements critical to its function. (**A**). The sequence of the C-terminal 69 residues of *E. coli* SSB is presented in the first line. The black line above the sequence corresponds to the putative polyproline type II helix, which terminates at residues 148 and 149 as these are known to disrupt these helices [72]. The red line demarcates the residues that form the linker domain. Enriched, the most over-represented residues highlighted in lines a and b. Residues 148 and 149 are highlighted to indicate where the PPII-helix would. Sequence analysis of the protein was done using REPRO at http://www.ibi.vu.nl/programs/ to determine the presence of repetitive elements. The spider silk sequence motifs that are repeated seven times are indicated. The location of the three PXXP motifs is highlighted by the red boxes. (**B**). Homology modeling reveals that residues 116–145 can adopt a collagen-like structure. The model of the N-terminal part of the SSB linker (green) is shown superimposed onto a collagen strand (purple).

How does binding between the linker domain and a partner OB-fold occur? The first insight into the mechanism of binding came from the analysis of the primary amino acid sequence [60]. This analysis revealed that this region of SSB is over-represented in glycine, glutamine and proline residues (Figure 3A and [64,67]). Several of these are arranged in repeats with as many as seven spider silk motifs. These are proposed to impart both flexibility and tensile strength to the linker, enabling SSB to bind to partners ranging in size from 20–80 kDa and to itself, to produce a stable complex on ssDNA. It was also shown that the N-terminal half of the linker can be modeled on a collagen strand further supporting the idea that these elements contribute to the flexibility of the linker (Figure 3B and [63]). Finally, and perhaps most importantly, the analysis revealed the presence of three, conserved PXXP motifs (Figure 3A, motifs highlighted by red boxes).

**Figure 4:**
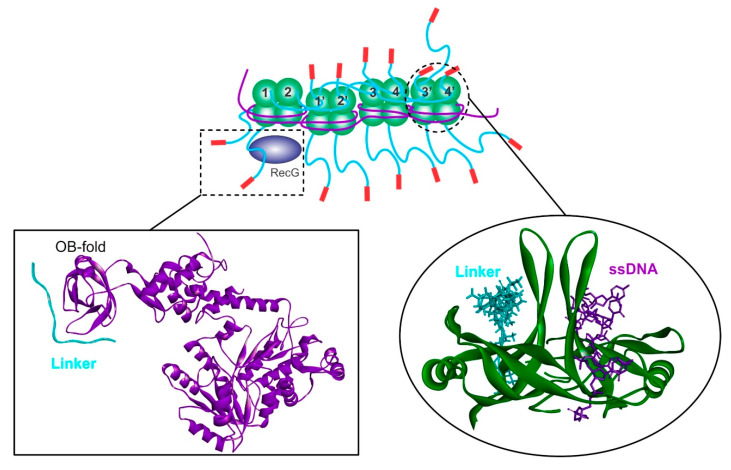
The linker/OB-foldnetwork of interactions is critical to SSB interactome function. Top, a schematic of an SSB-ssDNA complex demonstrating how the linker-OB-fold network of interactions produces a stable complex where the ssDNA is coated and protected. Here, the linker from the first tetramers bind to the OB-folds in adjacent tetramers, while other linkers are exposed and available for partner binding, in this case, RecG (left inset). Here, the helicase is presented as a ribbon diagram (purple) and a linker domain (cyan) is modeled into the OB-fold. When SSB-tetramers interact, some OB-folds are occupied by linker domains (cyan) while others bind ssDNA (purple). For clarity, only two monomers of the tetramer are shown and represented as ribbon diagrams (colored green). The schematic is adapted from [63].

In eukaryotic cells, PXXP-containing ligands bind Src homology 3 (SH3) domains to facilitate intracellular signaling [73,74,75,76]. Critically, SH3 domains are structurally, virtually identical to OB-folds with the folds aligning very well with an average root mean square deviation of less than 2.0 Å for the β-strands [77]. Importantly, OB-folds are present in both SSB and as many as twelve interactome partners [44,67,78,79,80,81,82]. Consequently, binding involves the docking of the linker of SSB into the OB-fold present in the partner protein (Figure 4 and [60,63]). When the interactions take place between the PXXP motifs in the linker of one SSB tetramer and one or more of the OB-folds in nearby SSB tetramers, cooperative ssDNA binding occurs (Figure 4, top panel). When these interactions occur between the linker-PXXP motifs of an SSB tetramer and the OB-fold in an interactome partner such as RecG, loading of that protein onto DNA takes place. It is not surprising then that mutations in key residues of the RecG OB-fold or, in the PXXP motifs of SSB, eliminate helicase binding and cooperative ssDNA binding, respectively [63].

This mechanism of binding has been termed the linker/oligonucleotide–oligosaccharide binding (OB)-fold interaction model or linker/OB-fold model [63]. Support for this model comes from several research groups who have demonstrated that the linker imparts species-specific, partner binding [67,68,83]. Finally, the binding site of alkylation protein B mapped to PXXP motifs II and III of SSB [69]. A caveat of this model is that linker and DNA binding to either SSB or partners is competitive and this has important implications for interactome function as explained below. As interactome members contain OB-folds, the SSB interactome has been classified as the first OB-fold family of genome guardians in *E. coli* [60].

### 3.2. RecG—The Regression Beast

RecG protein was identified as a mutation that mildly affected recombination and survival following UV-irradiation [84]. It was later shown that it participates in all three pathways of recombination and that it has an overlapping function with the products of the *ruvA* and *ruvB* genes [85,86]. Purified RecG has ATPase and DNA helicase activities [85,87]. It has been classified as a member of the SF2 DNA helicases and nucleic acid translocases [88].

The polarity of DNA uwinding by RecG unwinds is in the 3′ → 5′ direction [89,90]. It is active as a monomer on forks with either single-stranded or duplex arms as well as Holliday junctions [41]. RecG processes stalled replication fork substrates into structures that can be acted upon by additional members of the recombination machinery [24,80,91,92,93]. Furthermore, RecG exhibits significant ATPase activity on negatively supercoiled DNA, single-stranded DNA (ssDNA) and SSB-coated ssDNA [42,94]. This suggests different ways for RecG to access stalled replication fork. For example, the preference for negatively supercoiled relative to positively supercoiled DNA, suggests that DNA must first be acted upon by DNA gyrase for RecG to function [32,94,95,96].

The stimulation of ATPase activity on SSB-coated M13 ssDNA is intriguing and it was subsequently shown that a species-specific, protein–protein interaction between RecG and SSB is required [25,42,46]. This interaction is mediated through the linker domain of SSB and the oligonucleotide–oligosaccharide binding fold (OB-fold) in RecG (Figure 4; [60]). SSB-RecG binding is key to helicase function at a stalled fork since the enzyme can be directly loaded onto the DNA in the vicinity of single-stranded regions and is consistent with the role of SSB in targeting repair helicases to active forks in vivo [42,43,97].

**Figure 5:**
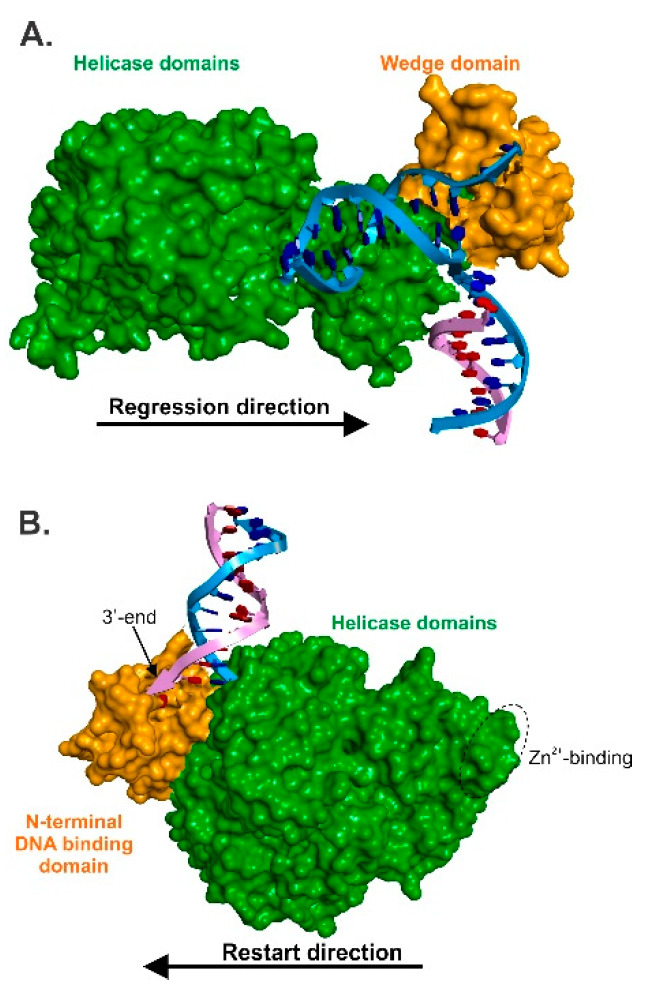
Replication fork rescue helicases. (**A**), RecG and (**B**), PriA. Each enzyme is represented as a Connolly surface. For RecG, a molecular model of the *Escherichia coli* enzyme is shown with the wedge domain colored orange and helicase domains in green. Adapted from reference [58]. For PriA, the N-terminal DNA binding domain is colored orange and the helicase domains are colored in green. The location of the zinc-binding sites is indicated by the dashed circle. Adapted from reference [82].

The crystal structure of the enzyme bound to a model fork substrate shows how RecG processes a fork [80]. The structure available is of the *Thermotoga maritima* protein. Except for the N-terminal extension whose function is unknown, the primary amino acid sequence is very similar to that of *E. coli* RecG (39% identical; [98,99]). Not surprisingly, homology models of *E. coli* RecG can be built using the *T. maritima* structure as a template (Figure 5A and [99,100]). The enzyme is divided into two general domains, highlighted in different colors. Domain I comprises of the N-terminal half of the protein and contains the wedge domain, which includes the oligonucleotide–oligosaccharide binding fold. The wedge domain is essential for specific binding to branched DNA structures and it is also intrinsic to DNA strand separation [101]. A long α-helical linker connects the wedge domain to the helicase domains. These C-terminal domains contain the helicase motifs, and couple the energy associated with ATP binding, hydrolysis and product release, to enzyme motion, DNA unwinding and rewinding and, fork clearing [80,100,102].

### 3.3. PriA—The Restart Specialist

Primosomal protein A (PriA) was originally known as factor Y and as protein n’. In vitro, the enzymeis required for the conversion of single-stranded ϕX174 DNA to the replicative form (reviewed in [103,104]). Here PriA binds to an ssDNA hairpin structure in ϕX174 called the n’-primosome assembly site (PAS), leading to the assembly of the primosome, a complex responsible for primer RNA synthesis and duplex DNA unwinding at a replication fork [104,105]. As the DNA sequence of PAS is unique, PriA can be considerd to be either a structure- or sequence-specific DNA binding protein, or both Following binding of PriA to PAS, the complex is then recognized and bound by PriB, PriC and DnaT. This is followed by the subsequent actions of DnaB, DnaC, DnaT and primase [103,104].

In addition to its role in phage DNA replication, PriA plays a crucial role in DNA replication fork rescue as shown in genetic studies [15,103,104,106]. Null mutations in *priA* result in a complex phenotype that includes constitutive induction of the SOS response, defects in the repair of UV-damaged DNA, DNA double-strand break repair and homologous recombination and these mutants exhibit defects in both constitutive and induced stable DNA replication [26,107,108,109,110]. Collectively, the indicate a key role for PriA in replisome assembly at sites distinct from *oriC* thereby facilitating replication restart [103,111,112].

The 82 kDa PriA DNA protein consists of two domains (Figure 5B and [113,114,115]). The N-terminal 181 aa is associated with DNA binding while the C-terminal 551 aa contains the ATP binding and DNA helicase motifs, which are interrupted by two, C4-type zinc finger motifs. These Zn-finger motifs are essential for in vitro primosome assembly on PAS, for recombination-dependent DNA replication in vivo and, for interactions with other primosomal proteins [116,117,118].

PriA binds to D-loops and to model, fork structures in vitro [119,120,121]. This binding is mediated through specificity of the N-termianl domain for DNA strands with accessible 3′-ends [115,122]. PriA belongs to helicase Superfamily 2 and has been shown to unwind DNA in the 3′ → 5′ direction [123,124]. DNA unwinding is fueled by the hydrolysis of ATP (dATP), is site-specific (i.e., PAS), structure-specific and ssDNA-dependent [125]. DNA unwinding of model fork substrates is stimulated by SSB and this involves both a physical and functional interaction between the two proteins [46,47]. Here, like that demonstrated for RecG, the OB-fold of PriA binds to the linker domain of SSB (Figure 4; [60]). This interaction is essential to the loading of PriA by SSB onto model forks substrates, which also similar to RecG and results in remodeling of the helicase [48].

Once loaded onto a stalled replication fork, PriA utilizes its helicase activity to unwind lagging-strand DNA present at the fork thereby generating a single-stranded DNA binding site for DnaB [120]. It is also responsible for loading of DnaB onto the exposed lagging-strand template [15,103,104]. Here it facilitates assembly of a multi-protein complex that includes PriB and DnaT. Intriguingly, while the helicase activity is required to generate ssDNA for DnaB, it is not required for the loading process itself [15,126]. During loading, the replicative helicase, DnaB, is transferred from a DnaB–DnaC complex onto ssDNA that can be exposed or SSB-coated. Once DnaB has been loaded, a new replisome forms, resulting in the resumption of DNA replication [9,103].

## 4. SSB-DNA Helicase Interactions during Loading

### 4.1. SSB-RecG

**Figure 6:**
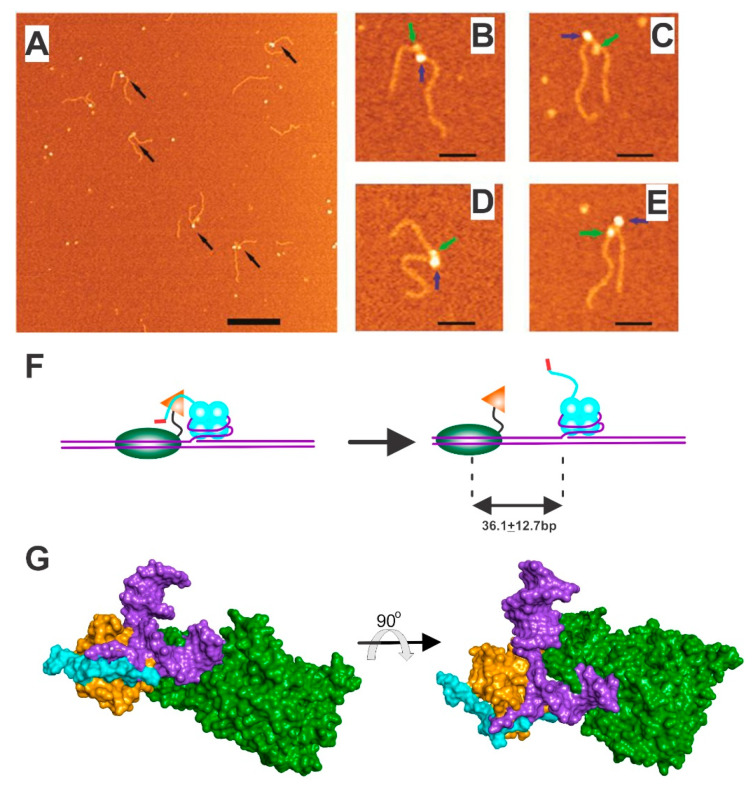
SSB loads RecG and remodels the helicase in the process. (**A**) Large scale atomic force microscopy image of SSB-RecG-DNA complexes. Proteins are indicated by arrows. The scale bar is 200 nm. (**B**–**E**), Zoomed images of four double-blob complexes. Large (SSB) and small (RecG) blobs are indicated with blue and green arrows (scale bars 50 nm). (**F**). A model for RecG loading by SSB. SSB binds to the fork first and RecG via the linker from one monomer within the tetramer. Loading ensues, concomitant with the remodeling of the wedge domain (orange) so that only the helicase domains (green) can bind DNA. Once loaded, RecG slides, using thermal energy, on average, 36 bp ahead of the fork. (**G**). Molecular models of RecG bound to a fork (purple) and the SSB linker (cyan; a PXXP-containing ligand). The wedge domain is colored orange and helicase domains in green to match the schematic in panel F. As fork and linker binding are competitive, the cyan and purple colors overlap demonstrating that cannot occupy the same space. The two images are rotated 90° relative to each other to enable clear visualization. AFM images are from [43].

It is known that SSB and RecG bind to one another both in vivo and in vitro [42,46]. Mechanistic insight into the dynamics of this interaction has been provided by atomic force microscopy (AFM) studies [43]. The preferred substrate for RecG, which is a fork with a gap in the leading strand, was used to visualize loading by SSB (Figure 6A and [41,42,127,128]). In these studies, SSB was bound to the fork first, followed by the helicase. RecG and SSB appear with different contrasts on AFM images, with SSB appearing larger than RecG by a factor of 2 [43]. Thus, SSB appears as a large blob and RecG as the smaller blob (Figure 6B–E; blue and green arrows, respectively). Further, SSB is always bound at the fork with RecG positioned on average, 36 bp upstream of the fork (Figure 6F). This positioning on the parental duplex region of the fork was the result of thermal sliding and was independent of a nucleoside triphosphate. Further evidence of sliding came from a study using high-speed AFM, where loading of RecG by SSB was followed by the helicase sliding on the parental duplex DNA [100].

During loading, the linker of fork-bound SSB binds to the RecG OB-fold (Figure 6G). As the wedge domain of the helicase is essential for fork binding, the only way that dsDNA binding followed by thermal sliding can occur is if the helicase domains are the only parts of the RecG associated with the DNA [101]. Therefore, when the linker of SSB binds to the RecG OB-fold, remodeling of the helicase occurs thereby enabling it to bind to and slide on, the parental duplex DNA. This sliding, which occurs in the presence and absence of ATP, plays an important role in fork rescue. If the parental duplex DNA near the fork is damaged or otherwise modified, RecG binding is impaired [24]. This may serve as a signal for RecG to disengage from the DNA, and for the repair machinery to repair the damage, before the onset of fork regression and/or replication restart.

### 4.2. SSB-PriA

A similar AFM approach was used to visualize PriA fork interactions and the effects of SSB on helicase loading. As ATP influences the overall structure of PriA in AFM, the studies described below were done in the absence of a nucleoside cofactor [48]. As for RecG, in the absence of SSB, PriA binds exclusively to forks but prefers a fork with a gap in the nascent leading strand. This result is in contrast to previous work, which demonstrated a preference for a fork DNA substrate with a gap in the nascent lagging strand [129,130,131,132]. This suggests that ATP may play an important role in dictating the specificity for PriA for fork substrates. This effect was not observed for RecG [43].

**Figure 7:**
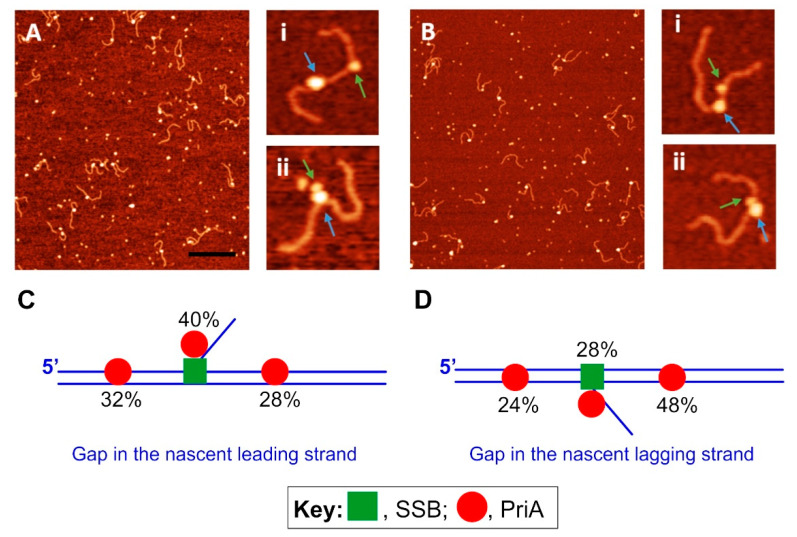
SSB loads PriA and remodels the helicase in the process. **A** and **B**, AFM images of PriA loading by SSB. The large blobs correspond to SSB (blue arrows) while the smaller ones correspond to PriA (green arrows). The insets in panels A and B are zoomed images. **C** and **D**, PriA locations following SSB loading and remodeling. The values represent the frequency of occurrence of PriA in each location. Adapted from reference [46].

Visualization of PriA loading by SSB revealed intriguing insight (Figure 7). First, SSB is always observed bound to the fork and appears as a large blob (blue arrows in insets Ai and ii; and Bi and ii). Second, and as a consequence of remodeling, PriA was observed as a small blob in distinct positions relative to SSB (green arrows in the insets). Furthermore, when the DNA substrate had a gap in the leading strand, PriA was loaded onto all fork arms by SSB with equal probability (Figure 7C). In contrast, when the substrate had a gap in the lagging strand, remodeled PriA was loaded preferentially to the leading strand (Figure 7D). These results suggest that like RecG, PriA was remodeled by SSB so that it could now bind to duplex DNA. This may facilitate the binding and/or translocation of PriA along the duplex in an ATP-independent manner. Surprisingly, and in sharp contrast to RecG, PriA was observed close to, or bound to, fork-associated SSB (Figure 7C,D). This was observed more frequently when the fork has a gap in the leading strand (40% of all complexes) than when the gap was in the opposite strand (28%; Figure 7C,D). These positions may correlate with SSB being “caught in the act” of loading, but this remains to be demonstrated unequivocally.

## 5. The Mechanics of Fork Regression by RecG

Once loaded at a stalled replication fork, RecG regresses the fork away from the site(s) of DNA damage. In this reaction, the nascent leading and lagging arms are unwound and this is coupled to rewinding of the parental duplex and the middle toe of the chicken foot (Figure 1A). This requires a specialized DNA helicase that must have the ability to couple unwinding of the nascent leading and lagging strand arms to duplex rewinding (Figure 1B). Additionally, this specialized DNA helicase must be able to generate sufficient force to clear the fork of potentially numerous proteins such as SSB (Figure 1C).

**Figure 8:**
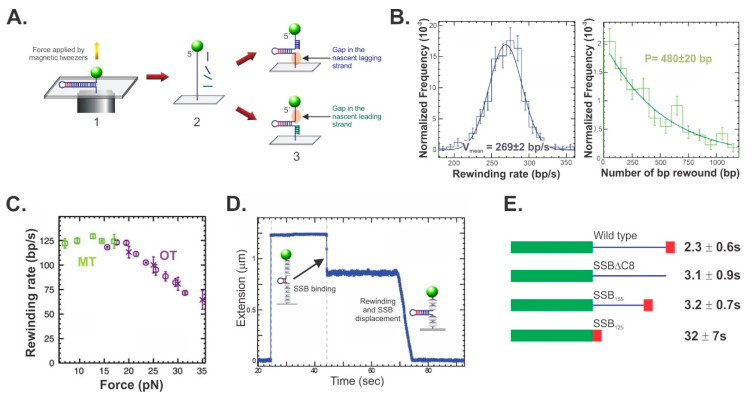
RecG catalyzes the key steps required for fork regression and displaces SSB in the process. (**A**) Construction of forks with gaps in either the leading or lagging strands. Here, the 1200 bp hairpin is fully stretched by the application of force from the magnetic tweezers. Then, oligonucleotides complementary to the 5′- or 3′-proximal regions are introduced in separate reactions and allowed to bind. Once the opposing force is decreased, a partial hairpin is extruded and as the oligonucleotide remains annealed to reveal a fork with a gap on the opposite side, either the lagging (top) or leading strand (bottom). (**B**), RecG rewinds forks with rates and processivity values consistent with a distance-limited fork regression reaction. The reactions rates and processivity of RecG-catalyzed fork rewinding at 37 °C were obtained using the hairpin substrate shown in panel A. P, processivity. (**C**), RecG can work against large opposing forces during fork rewinding. MT, data from magnetic tweezer experiments. OT, data from assays using optical tweezers; (**D**), RecG readily displaces SSB during fork rewinding. Here, the hairpin is fully unwound by the application of force. When SSB is added, binding results in the wrapping of the polynucleotide around the tetramers. This causes a shortening of the DNA tether. (**E**), Functional linkers are required for SSB displacement by RecG during fork rewinding. Each SSB is colored green (N-terminal domain), blue (linker) and red, acidic tip. SSB_155_ and SSB_125_ are mutant proteins where the linker was either partially or completely removed, respectively. The values next to each protein, represent the lag time for rewinding to initiate following the addition of RecG. Data panels B-D are from [24] and panel E is from [135].

Single-molecule studies using magnetic tweezers revealed that this enzyme is the RecG DNA helicase [24]. Using a 1200 bp hairpin, RecG was shown to rewind complementary fork arms; to couple DNA unwinding to this rewinding and to generate Holliday junctions in the process (Figure 8). Importantly, RecG catalyzed fork regression only. During the coupled unwinding/rewinding reaction, that is fork regression, movement of the fork proceeded at a rate of 269 ± 2 bp/sec and with a processivity of 480 ± 20 bp. As RecG utilizes 1 ATP to translocate 3 bp, 160 nucleoside triphosphate molecules are hydrolyzed on average per fork regression event [35,133]. By using a combination of both magnetic and optical tweezers, the authors demonstrated that RecG could catalyze rewinding against forces of up to 35 pN, with only a moderate drop of about 40% in rate (Figure 8C). This is consistent with RecG being a very powerful motor that should be able to clear the fork of bound proteins. This was tested using SSB, which requires at a minimum, 10 pN of force per tetramer [134]. Not surprisingly, the protein posed little threat to RecG as the enzyme was capable of coupling rewinding to efficient SSB displacement (Figure 8D). Subsequent studies determined that the SSB linker is required for efficient displacement as mutant SSBs with the linker either partially or completely deleted, significantly impaired the ability of RecG to displace them (Figure 8E). This does not require linker/OB-fold interactions as the RecG OB-fold is already bound to the fork. Instead, this reflects the ability of RecG to push SSB off the DNA. When linker/OB-fold, SSB–SSB interactions were in play as they were for the wild type and SSBΔC8, the pushing by RecG was communicated between the SSB tetramers facilitating displacement. In contrast, when SSB–SSB linker/OB-fold interactions were absent, the SSB_125_ tetramers, which have no linker, functioned as separate, tightly bound entities that impede RecG translocation.

## 6. Summary

Stalled DNA replication fork rescue is essential. It requires physical and functional interactions between the SSB protein and the DNA helicases, PriA and RecG.SSB-helicase binding is critical to the loading of these enzymes onto stalled forks. During the loading process, each helicase is remodeled by SSB. For RecG, the wedge domain is bound to the linker of SSB and cannot gain access to the fork. Consequently, the helicase domains of RecG mediate DNA duplex binding followed by thermal sliding ahead of the fork. This permits theclearing of bound proteins in its immediate vicinity as well as tesing of the integrity of the DNA. Once RecG is loaded, it catalyzes an efficient regression reaction in the presence of ATP. Here, the enzyme works against large opposing forces, coupling fork rewinding to the displacement of tightly bound proteins such as SSB. RecG is unique in its fork function, catalyzing fork regression only producing Holliday junctions for subsequent processing by additional enzymes such as RuvAB. Once this additional processing has taken place and the original fork structure restores, PriA is loaded onto the DNA, resulting in the ultimate reloading of the replisome. For PriA, the N-terminal DNA binding domain is also bound by the linker domain of SSB during loading. Consequently, the helicase is loaded onto the fork arms with the final location dictated by the presence of single-stranded DNA in the opposite arm. Similar to RecG, this binding is mediated by the helicase domains of PriA. Collectively, the interactions between SSB and these two critical helicases result in the restoration of the fork and the restart of DNA replication.
